# 

**Published:** 1858-10

**Authors:** 


### Journal of Public Health.

INCLUDING THE

### TRANSACTIONS OF THE EPIDEMIOLOGICAL SOCIETY OF LONDON.

EDITED BY

BENJAMIN W. RICHARDSON, M.D.,

LICENTIATE OF THE ROYAL COLLEGE OF PHYSICIANS,
PHYSICIAN TO THE ROYAL INFIRMARY FOR DISEASES OF THE CHEST, AND LECTURER
ON PUBLIC HYGIENE AT THE GROSVENOR PLACE MEDICAL SCHOOL.

NATIONAL HEALTH] IS NATIONAL WEALTH.

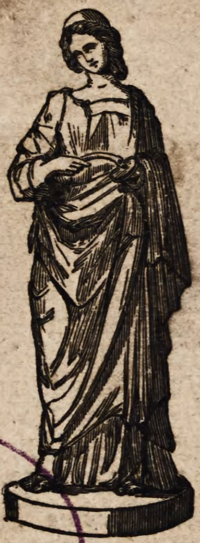

Ὑγίεια
πρεσβίστη μακάρων.

ARMY
MEDICAL
OCT 1 - 1946
LIBRARY

LEES-MUSEUM
BIBLIOTHEEK
TE AMSTERDAM.

PRINTED AND PUBLISHED FOR THE PROPRIETOR BY

THOMAS RICHARDS, 37, GREAT QUEEN STREET.

1858.

[Published Quarterly.—Annual Subscription, 10s. Payable in Advance.]

